# Using Machine Learning to Automate the Analysis of an Olfactory Habituation‐Dishabituation Task in Mice

**DOI:** 10.1002/brb3.71619

**Published:** 2026-07-28

**Authors:** S. Boyanova, M. H. Correa, R. S. Bains, F. K. Wiseman

**Affiliations:** ^1^ UK Dementia Research Institute at University College London London UK; ^2^ Mary Lyon Centre, Medical Research Council Harwell Oxfordshire UK

**Keywords:** automation, mouse behaviour, olfaction

## Abstract

**Introduction:**

Improving the efficiency and accuracy of annotation and extraction of performance data from mouse behavioral tasks will improve both the throughput and scientific value of preclinical research.

**Methods:**

Here, we present and validate an automated pipeline for the annotation and quantification of performance in a mouse olfactory habituation‐dishabituation task, using a single side‐view camera, resulting in occluded body parts. We created a pipeline for task analysis, combining DeepLabCut (DLC) for pose‐estimation and SimBA, for behavioral classification to automatically quantify odor interaction (sniffing time) in a three‐odor (water, familiar mouse social odor, novel mouse social odor) variant of the task. We used a subset of previously published, fully manually annotated datasets to train the models and unseen videos from the same study to validate the utility of our machine learning (ML) pipeline.

**Results and Conclusion:**

Our analysis pipeline estimated behavioral performance in the task with high accuracy, and the data produces similar technical and biological results to manual methods when analyzed by linear mixed modeling. Thus, we validated the utility of our customized pipeline for the automated scoring of this mouse sensory task, and we discuss its strengths and limitations.

## Graphical abstract

Workflow of DeepLabCut and SimBA pipeline for analysis of olfactory habituation‐dishabituation task in mice.

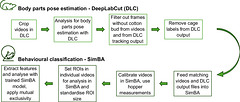

## 1 Introduction

Behavioral analysis is fundamental to the assessment of preclinical mouse models of neurological disease (Von Ziegler et al. 2021). Sensory inputs and motor outputs are critical to the manifestation of behavior and cognitive processes and can be independently impacted by disease (Ison et al. 2007; Rodgers et al. 2012; Ryan et al. 2008). Thus, the battery assessment of both clinically relevant behaviors/cognitive domains and associated sensory and motor processes is important for the assessment of new lines and determining the potential translational impact of interventions. Olfaction is a key sensory input in mice and is particularly important for the interpretation of social tasks, including the Crawly three chamber test of social motivation and memory (Ryan et al. 2008). Olfaction can be quantified in mice using the habituation‐dishabituation task to test the animal's ability to differentiate between different odors by measuring the amount of time an animal interacts with (sniffs) repeated presentation of a series of odors. Novel odors typically elicit increased interaction, which wanes (habituates) over time.

Recent machine learning (ML) methods to automate the quantification of behavior permit a dramatic increase in analytical throughput and accurate measurement of a greater range of behaviors. A wide variety of ML tools capable of body parts pose estimation, such as DeepLabCut (DLC) (Mathis et al. 2018; Pereira et al. 2022), and behavioral classification (Chan et al. 2025; Marks et al. 2022), such as SimBA (Goodwin et al. 2024), have been developed. DLC is a pose‐estimation framework based on deep convolutional networks commonly including ResNets, pretrained on the large‐scale object recognition benchmark ImageNet. The ResNet outputs spatial probability densities, the probability density of each body part represents the ‘evidence’ that this body part is at a particular location (Mathis et al. 2018). To then generate meaningful behavioral quantification, the outputs from DLC are further processed using a ML behavioral classifier to determine the duration or frequency of behaviors of interest. The ML tool used to train behavioral classifiers in this study, SimBA, uses random forest ML classifiers for behavioral predictions by computing explainable feature representations of movement, angles, paths, velocities, distances, and sizes within individual frames and as rolling time window aggregates (Goodwin et al. 2024).

The most frequent use case of such ML tools for mouse behavioral analysis focuses on top‐view tracking, for example, SimBA (Goodwin et al. 2024). However, in some tasks, much of the complexity of animal behaviors is better captured using a side‐view projection, for example, the interaction of an animal with odor cues in the olfaction habituation‐dishabituation task performed in an individually ventilated cage (IVC). Importantly, the use of a single side‐view camera in a complex environment such as the IVC creates a technical challenge caused by the frequent occlusion of body parts, and difficulties with depth estimation. Furthermore, it is an established bottle neck in the field that the DLC output of body part coordinates can be noisy and challenging to process (Biderman et al. 2024; Klecel et al. 2025), especially in complex settings often found in animal science, like barn walkways or clinical exams (Klecel et al. 2025), but also in the laboratory IVC setting. This often requires additional video preprocessing, DLC coordinate post processing (Klecel et al. 2025), and network customization (Panconi et al. 2025). Here, we selected two of the most mature and validated ML tools in the behavioral community—DLC (Mathis et al. 2018) and SimBA (Goodwin et al. 2024) to develop a customized video preprocessing and automated scoring pipeline for the olfaction habituation‐dishabituation task filmed from the side with a single camera in an IVC in the presence of sawdust bedding and a food hopper. We present and validate this customized pipeline for the automated quantification of odor cue interaction (sniffing) using single side‐view videos of the habituation‐dishabituation task in laboratory mouse models of human disease. We used a manually annotated (ground‐truth) dataset of mice undertaking the olfaction habituation‐dishabituation task as part of a previously published longitudinal test battery (Boyanova et al. 2025). This study used two independent lines of genetically modified animals that model aspects of ALS/FTD (*C9orf72^GR400/+^* and *Tardbp^Q331K/Q331K^*). This pipeline can be used or adapted to automate the quantification of the olfaction habituation‐dishabituation task using a side‐view projection of odor cue presentation, facilitating rapid and accurate data production for the study of other animal models of disease and fundamental biology.

## 2 Methods

Primary data for this paper were previously published in Disease Models and Mechanisms (Boyanova et al. 2025), methods for data acquisition are reported below.

### 2.1 Animal Welfare and Husbandry

All animals were housed and maintained in the Mary Lyon Centre at MRC Harwell under specific pathogen‐free (SPF) conditions, in IVC adhering to environmental conditions as outlined in the Home Office Code of Practice. All animal studies were licensed by the Home Office under the Animals (Scientific Procedures) Act 1986 Amendment Regulations 2012 (SI 4 2012/3039), UK, and additionally approved by the Institutional Ethical Review Committees. Mice were randomized, blocked by genotype and sex at the time of weaning, into cages of 3–5 mice. All mice used in the study were bred in the Mary Lyon Centre at MRC Harwell and were housed in IVCs (Tecniplast BlueLine 1284), on grade 4 aspen wood chips (Datesand, UK), with shredded paper shaving nesting material and small cardboard play tunnels for enrichment. The mice were kept under controlled light (light 07:00–19:00; dark 19:00–07:00), temperature (22°C ± 2°C), and humidity (55% ± 10%) conditions. They had free access to water (25 p.p.m. chlorine) and were fed ad libitum on a commercial diet (SDS Rat and Mouse No.3 Breeding diet (RM3)).

### 2.2 Animal Genetics

The generation of the *C9orf72^em2.1Aisa^* (MGI:6827370), here called the *C9orf72^GR400/+^* mouse model, is described in Milioto et al. 2024 (Milioto et al. 2024). The *C9orf72^GR400/+^* line was maintained on a C57BL/6J background by heterozygote backcross before the generation of phenotyping cohorts. Phenotyping cohorts were generated by crossing either male or female *C9orf72^GR400/+^* heterozygotes to C57BL/6J wildtypes (WT). The generation of the *Tardbp^em1Rhbr^* (MGI:6157626), here called the *Tardbp^Q331K/Q331K^* mouse model is described in White et al. 2018 (White et al. 2018). The *Tardbp^Q331K/Q331K^* line was maintained on a C57BL/6J background by heterozygote backcross prior to the generation of phenotyping cohorts. Phenotyping cohorts were generated by heterozygote intercross to generate WT and *Tardbp^Q331K/Q331K^* homozygote animals.

### 2.3 Genotyping

DNA was extracted from ear biopsy, isolated at postnatal day (P)14 using TaqMan Sample‐to‐SNP (Applied Biosystems). Mice were genotyped for *C9orf72^GR400^* using TaqMan WT and mutant quantitative PCR assays duplexed with the *Dot1l* reference allele. The following primers and probes were used for the *C9orf72^GR400^* mutant allele (forward, 5′‐TTCCAGATTACGCTTACCATAC‐3′; reverse, 5′‐CGACCTCTTCCTCGTCCT‐3′) and probe (5′‐FAM‐TACCTCGTCCACGTCCTCGTCTTC‐BHQ1‐3′), *C9orf72^+^* WT allele (forward, 5′‐CTATTGCAAGCGTTCGGATAATG‐3′; reverse, 5′‐CTTGGCAACAGCAGGAGAT‐3′) and probe (5′‐FAM‐TGGAATGCAGTGAGACCTGGGATG‐BHQ‐3′), and reference *Dot1l* allele (forward, 5′‐TAGTTGGCATCCTTATGCTTCATC‐3′; reverse, 5′‐GCCCCAGCACGACCATT‐3′) and probe (5′‐VIC‐CCAGCTCTCAAGTCG‐MGBNFQ‐3′). Mice were genotyped for the *Tardbp^Q331K^* alleles using allelic discrimination assays, with a common pair of primers for both *Tardbp* alleles (forward, 5′‐TCTGCTGGCTGGCTAACAT‐3′; reverse, 5′‐GGGTGGAGGGATGAACTTTG‐3′). To discriminate between the mutant and WT alleles, different probes were used (*Tardbp^Q331K^*, 5′‐TET‐AACTGCTCTTCAACGCT‐BHQ1‐3′; *Tardbp^+^*, 5′‐FAM‐CAACTGCTCTGCAACG‐BHQ‐3′).

### 2.4 Olfaction Task

The olfaction test was run at 15‐ and 67‐weeks of age. The number of mice in the *C9orf72* study at 15‐weeks of age was WT = 24 (female = 12, male = 12), *C9orf72^GR400^* ^/+^ = 24 (female = 12, male = 12); at 67‐weeks of age WT = 20 (female = 11, male = 9), and *C9orf72^GR400^* ^/+^ = 18 (female = 9, male = 9), where two videos were excluded due to the wrong food hopper being used for the task due to a technical error. In the *Tardbp* study at 15‐weeks of age—WT = 27 (female = 12, male = 15), *Tardbp^Q331K^* ^/^ *^Q331K^* = 24 (female = 9, male = 15), where three mice were excluded due to the wrong order of odor presentation during the test and were treated as a procedural failure. At 67‐weeks of age—WT = 21 (female = 11, male = 10), *Tardbp^Q331K/Q331K^* = 19 (female = 10, male = 9), where one mouse was excluded due to corrupted video file.

Mice were removed from their home cage and allowed to acclimatize to a clean IVC with clean sawdust bedding and an empty food hopper placed in a Home Cage Analyser system (Actual Analytics Ltd., Edinburgh, UK) for 30 min prior to the start of the test. During this period, the mice had no access to food but had access to water. We used a Flea 3 infra‐red camera (monochrome, pixel size 5.3 × 5.3 µm), which is part of the Home Cage Analyser system (Actual Analytics Ltd., Edinburgh, UK). The videos were recorded at a resolution of 1280 × 720 pixels at 25 frames per second (fps). The odors used for this test were water (control), familiar mouse, and novel mouse (social odors) presented on sterile cotton buds through the access for the water bottle of the IVC. The cotton buds for water were prepared by pipetting 50 µL of deionized water onto the swab. The social odors were prepared by wiping the cotton bud in a zigzag fashion across the bottom of a used cage, either the animal's home cage or an unfamiliar cage. After 30 min, the water bottle was removed, and the first cotton bud was presented in the cage through the water bottle access with the food hopper in place. The mouse was allowed to explore the cotton bud freely for 2 min, followed by 1 min inter‐trial interval. Each odor type was presented three times in a row. The order of the odor types was counter‐balanced and randomized across the mouse cohorts. The time spent sniffing each cotton bud was scored manually using Simple Video Coder (Barto, Bird, Hamilton, and Fink 2017), the scorer was unaware of the mouse genotype. Sniffing was defined as the orientation of the mouse's head and nose towards the cotton bud, and the distance of the nose at least 1 cm away from the front and the bottom of the food hopper, as well as the lower one third of the back of the food hopper. Licking or biting of the cotton bud was not included in the time scored. Thus, the task was set up to prevent physical access to the cotton bud presenting the odors, and it assesses the habituation and dishabituation to the volatile components of the odours but not the non‐volatile ones, such as non‐volatile pheromones (Liberles 2014; Noack et al. 2010). If the mouse spent less than 10 s interacting with the first presentation of a given odor, this trail and the consecutive two presentations of the same odor were not used in the analysis, since the mouse failed to sufficiently engage with the stimulus on the first presentation (‘Behavioral Models | Behavioral and Functional Neuroscience Lab | Wu Tsai Neurosciences Institute’ 2023). We implemented this task engagement cut‐off in order to avoid floor effects which have been observed in previous reports when mice are presented with odors that are similar to each other or with multiple novel odors (Arbuckle et al. 2015; Yang and Crawley 2009), also this approach can be used to estimate the level of engagement with the task.

### 2.5 Machine Learning Pipeline Training

#### 2.5.1 Video Preprocessing for DeepLabCut Training

**FIGURE 1:**
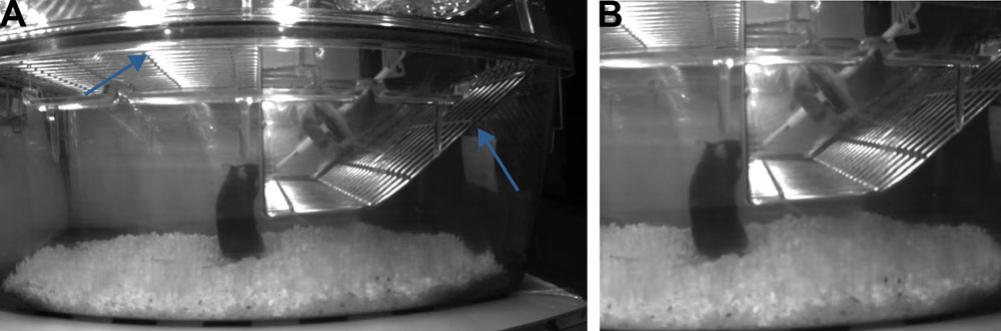
Preprocessing of videos of the olfactory habituation‐dishabituation task. (A) Full frame from an example video; and (B) cropped frame used for training and validation on unseen videos in the pipeline. Blue arrows show the hopper bars used as borders to crop the area around the hopper consistently between the videos.

For the DLC training stage, a total of 60 videos were selected, and balanced for age and genotype – 15 videos per age per genotype from each study (WT and *C9orf72^GR400/+^*, WT and *Tardbp^Q331K/Q331K^*), from a total of 183 videos from the study, previously analyzed using manual annotation (Boyanova et al. 2025). To avoid bias, the experimenter was unaware of the genotype during this process. The original format of the olfaction videos was .flv, and all videos were converted to .mp4 format using the format conversion tool in Simple Behavioral Analysis (SimBA v 2.0.7). Then the DLC v. 2.3.9 video editor tool was used to crop the videos to the region of interest (area proximal to the metal hopper where the odor‐cotton buds are presented) (Figure 1). This reduces the size of the videos and the complexity of animal behavior recorded, speeding up downstream processing.

#### 2.5.2 Pose Estimation Training Using DeepLabCut

To track the position of the animal, DLC v. 2.3.9 software (Mathis et al. 2018) was trained using the preprocessed training set of 60 videos, Nvidia RTX4090 GPU was used during training.

**FIGURE 2:**
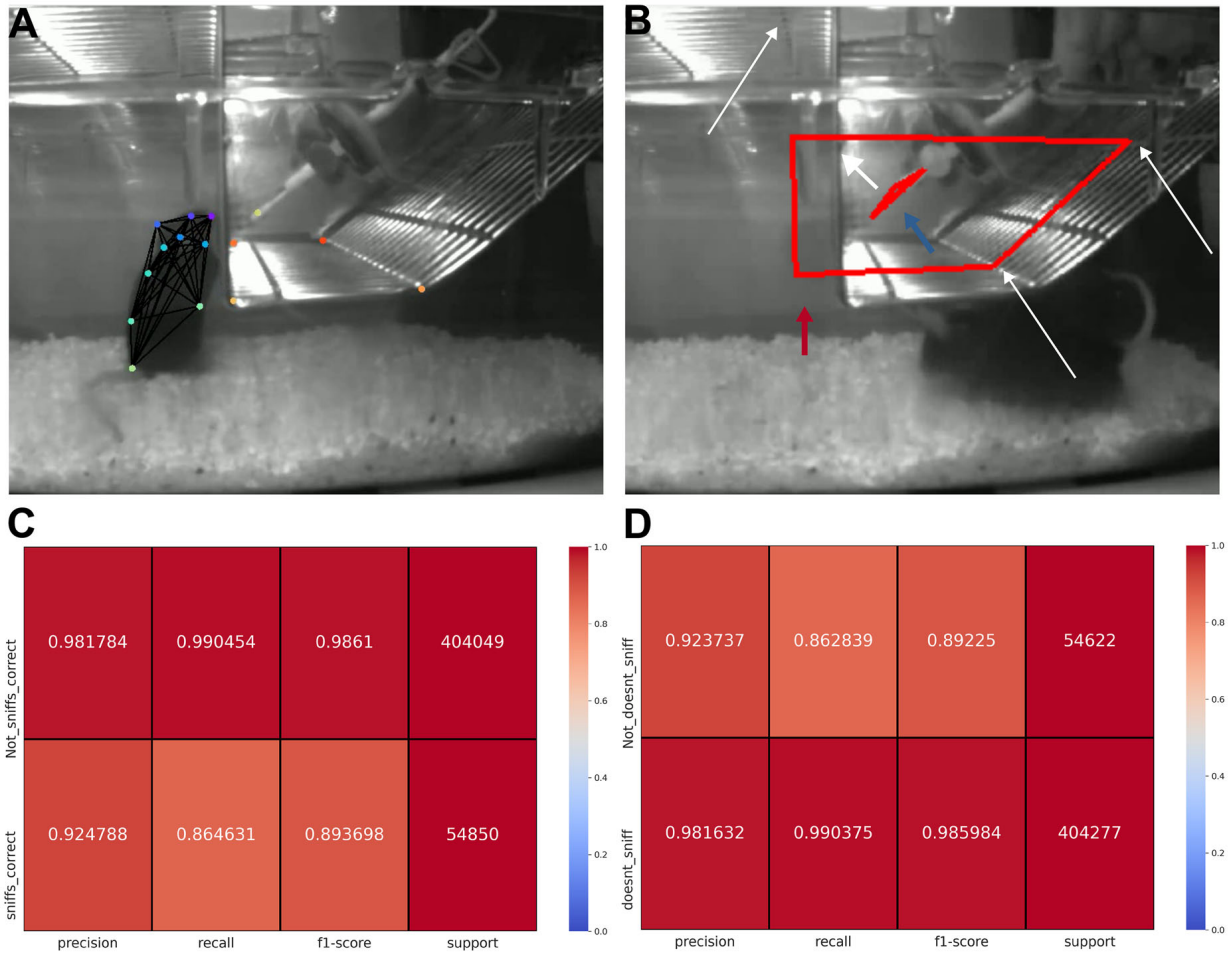
Machine learning annotations for the olfactory habituation‐dishabituation task analysis pipeline. (A) DLC labelling of mouse body parts (green, blue, and purple dots), tip of cotton bud presenting odour cue (yellow) and hopper landmarks (red and orange); (B) SimBA ROI positioning for training of hopper (red arrow) and cotton bud (blue arrow) aligned by food hopper bars shown by white arrows; (C); and (D) precision, recall, F1 score, and support assessment of the SimBA trained models for the presence and absence of sniffing behaviour.

The final model included 10 body parts of interest on the mouse (snout, middle point at eye level, left ear, right ear, back of the neck, front of the neck, two points at the back, base of the tail, and abdomen), additional six spatial locations relevant to the behavior of interest were also annotated (four corners of the hopper, the tip of the cotton bud, the checkerboard floor of the arena when visible) (Figure 2A).

The model was developed through an iterative training and refinement procedure over 16 cycles. These included initial exploration of resnet_50 and resnet_101 backbones without the key points for the front of the neck and the checkerboard. Following these two initial evaluations, the model was retrained from scratch with resnet_101 and further key point incorporation for the front of the neck and the checkerboard to improve tracking accuracy. This configuration of the model was then refined through an iterative process, incorporating and additionally labeling frames from the set of 60 videos, selected through outlier detection within DLC and manual inspection of ML labeling mistakes. Manually extracted additional frames focused on instances where the nose of the mouse was showing between the bars of the hopper, as these proved to be the most difficult for the model to label correctly. Between 18 and 336 additional frames were added in consecutive cycles, labeled and incorporated into the training dataset, the model was then retrained from previously trained weights after dataset expansion. We used a batch size of 8, and imgaug image augmenter. For both training and retraining, the dataset was split randomly (shuffle = 1) into train (95% of frames) and test (5% of frames) subsets to train and assess network performance, respectively, for each cycle. The p cut‐off was set to 0.6. Test error—mean absolute error measured in pixels between the predicted position of the labels and the manual annotation was used to assess network performance. The training cycles were repeated until a train error of 1.84 px and a test error of 2.42 px was reached (spatial resolution of test error approximately 1.1 mm). When applying the p‐cutoff (0.6), the train error was 1.78 px, and the test error was 2.1 px. The final model was trained on 2069 labeled frames from the 60 videos training dataset, with 2,150,000 iterations. It was selected based on the convergence of training and test errors, as well as visual assessment of the tracking accuracy on a subset of unseen videos (*n* = 16 videos). Following this step, the model was used for the analysis of the full unseen dataset.

#### 2.5.3 Post Processing of DLC Coordinates and Videos for SimBA Training

To define sniffing of introduced odor cues, Simple Behavioral Analysis was used (SimBA v 2.0.7) as per published guidelines (Goodwin et al. 2024). The SimBA model was trained on 80 videos in total, this included the 58 videos used for DLC training, two were excluded from the SimBA training due to an acquisition technical error of wrong order presentation of odor (see Supplementary Table 1). The videos were processed for SimBA training by keeping only frames that contained the cotton bud (DLC likelihood of 0.8 or higher). The cotton bud, cage, and checkerboard pose estimation data for these frames were subsequently removed from the DLC output before feeding into SimBA. Removing frames without cotton bud presentation reduced the size of the dataset and improved the balance between “sniffs” and “does not sniff” behavioral classifiers of interest, improving the accuracy of classifications.

#### 2.5.4 Behavioral Classifiers Training Using SimBA

The videos, and DLC pose estimation data containing only frames with a cotton bud likelihood of 0.8 or higher and only coordinates for the mouse body parts were fed into SimBA (Figure 2A, blue, green, and purple dots). SimBA annotated regions of interest (ROIs)—metal hopper and cotton bud were also applied (Figure 2B). This was combined with manual scoring of sniffing performed in Simple Video Coder, transformed to Solomon format using custom‐made Python scripts, and imported into SimBA (all data and scripts available on GitHub sboya23/ML‐analysis‐olfaction). Within the SimBA workflow, the DLC body pose estimation data was used without outlier correction, and manual scoring data was used to train SimBA to recognize two behavioral classifiers—“sniffs” and “does not sniff”.

The ROIs (metal hopper and the cotton bud) were applied to aid feature extraction in each frame (Figure 2B). For the training phase, the ROIs were set in a single video, then applied to all videos in the training set, the size of the ROIs was standardised according to the first video in which the ROIs were set. Feature extraction, ROI data appended to the features extracted by body part, and extra sub‐feature extraction (within animal three body part angles and frame by frame body part movement) were used to train “sniffs” and “does not sniff” behavioral classifier models. We used 201 features computed in SimBA within individual frames and as rolling time‐window aggregates of 66–500 ms (Goodwin et al. 2024). For the list of features used, see feature importance log files ML‐analysis‐olfaction/SimBA‐training at main · sboya23/ML‐analysis‐olfaction. The models were trained with the following settings—random forest algorithm with 2000 estimators, the frames were split into training and test set, and 20% of the data was reserved for testing model performance (train‐test split type—frames, test size – 0.2). The training was performed with two learning curve splits (see ML‐analysis‐olfaction/SimBA‐training at main · sboya23/ML‐analysis‐olfaction). The models showed precision = 0.92, recall = 0.86, and F1 score = 0.89 for “sniffs”, and precision = 0.98, recall = 0.99, and F1 score = 0.98 for “does not sniff” (Figure 2 C and D). When using the trained model for analysis, the optimized discrimination threshold for “sniffs” and “does not sniff” was 0.5, with 200 ms duration of the behavioral bout.

#### 2.5.5 Validation of the Model on Unseen Videos

To validate the devised pipeline, the trained DLC and SimBA models were used to determine sniffing times in a set of unseen test videos that had been previously manually scored (Boyanova et al. 2025). The videos were preprocessed for DLC analysis as during the training phase and analyzed using the trained DLC model for pose estimation. The DLC output and the tracked videos were post processed as already described during the training phase. The body parts output was fed into a new SimBA project. SimBA ROIs were positioned for each video, the ROI size was standardized according to a randomly selected video, and features were extracted as during the training phase. The previously trained SimBA models classified both “sniffs” and “does not sniff” bouts (0.5 discrimination threshold and 200 ms bout length), mutual exclusivity of “sniffs” and “does not sniff” was applied, where “sniffs” was set as the winner with threshold 0 to ensure mutually exclusive classifications in favor of “sniffing”. The time spent sniffing was determined by the model in 1 s time bins, which were then subdivided for the nine odor presentations into time periods of interest for each video to generate the time spent sniffing of each odor cue (three odors presented three times for an equal amount of time adjusted to the individual length of each video).

#### 2.5.6 Statistical Analysis

Pearson's r and R^2^ were calculated in R Studio (R version 4.4.2) to measure the correlation between manual and ML scoring of videos and between two manual scorers. Generalized linear mixed effects models (glmer) and linear mixed‐effects models (lmer) and the lme4 and lmerTest packages (Bates et al. 2015; Kuznetsova et al. 2017) in R Studio (R version 4.4.2) were used for data analysis, based on (Colom‐Cadena et al. 2023; ‘Spires‐Jones Lab GitHub’ 2025). To assess if the manual scoring and the ML method were estimating task engagement differently, we used a glmer model with a binominal distribution: status ∼ source * age with individual video as a random effect, where status means whether a video would be filtered for the 10 s engagement cut‐off or not (0, 1), and source—whether the video was scored using the manual or ML method. If the mouse spent less than 10 s interacting with the first presentation of a given odor, this trial and the consecutive two presentations of the same odor were filtered out. We performed an analysis of the effect of age using the average age of the mice at every time point; thus, we had two age groups for each study (*C9orf72* versus WT controls and *Tardbp* versus WT controls). Fixed factors and interactions (here denoted by *) were assessed using ANOVA Type III Wald chi square tests.

To assess whether there is a significant difference in the values obtained from manual or ML scoring in mice engaging with the task, and to assess the effects of genotype, odor type presentation, and sex, we used the lmer model time spent sniffing ∼ source*genotype*(odor type presentation + sex) with the individual video as *a random effect* for both studies. Here, and throughout the paper * denotes assessment of fixed effects and interaction between them. Odor type presentation was defined as all three presentations of each odor—water, familiar mouse and novel mouse odor, and the two age groups were analyzed separately for each mouse model (*C9orf72* versus WT controls and *Tardbp* versus WT controls). Factor significance was assessed using Type III ANOVA with Satterthwaite's method and is reported (Colom‐Cadena et al. 2023). For this task, analysis of performance can only be measured if sufficient engagement with the odor cues occurs at the first presentation; thus, data must be filtered for the 10 s cut‐off to remove experiments that do not meet this requirement. Diagnostics for glmer and lmer were performed by applying the DHARMa diagnostics package (Hartig et al. 2024), and residuals plotting, respectively. The emmeans package with Bonferroni correction was used for post hoc analysis of comparisons of interest when a significant main effect of a variable was observed to identify which groups differed. Python and R script generation and refinement were assisted by the large language model ChatGPT.

## 3 Results

### 3.1 Correlation Between Manual and ML Sniffing Time in the Olfactory Habituation‐Dishabituation Task

To determine the efficacy of our ML pipeline to quantify the time spent interacting with odor cues (sniffing) in the olfactory habituation‐dishabituation task, we performed correlation analysis between manual scoring and the output of the ML analysis pipeline. We evaluated our pipeline using previously reported data (Boyanova et al. 2025) comparing colony‐matched WT and *C9orf72^GR400/+^* mice, and colony‐matched WT and *Tardbp^Q331K/Q331K^* mice that model different aspects of ALS/FTD, and which were manually scored. These data are longitudinal and measured olfaction in young (15‐weeks of age) and old (67‐weeks of age) mice.

**FIGURE 3:**
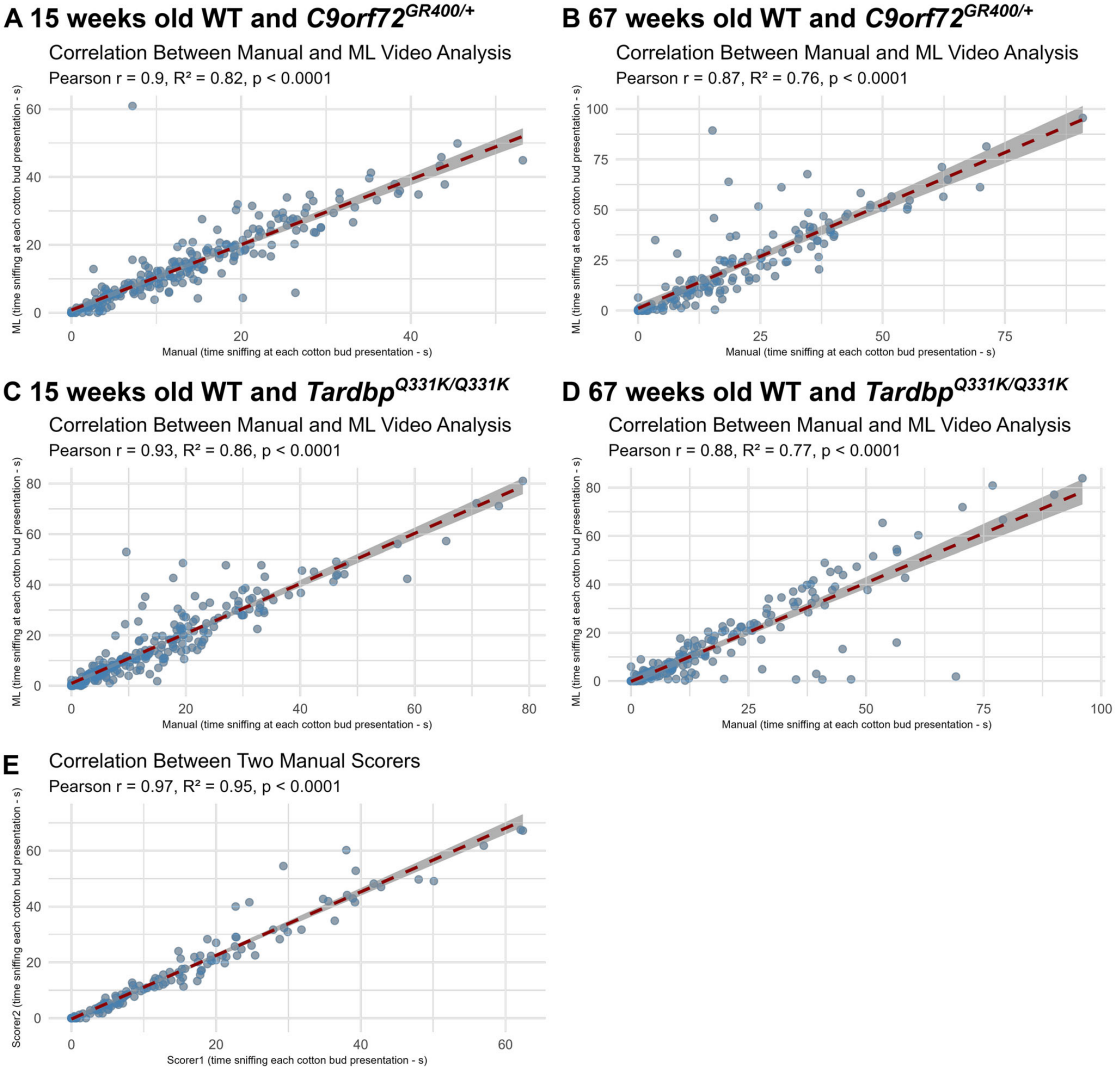
Relationship between sniffing time estimation by manual annotation and automated ML pipeline. Correlation between manual and ML estimation of the time spent sniffing (s) each odour presentation in (A) 15‐weeks of age (Pearson's *r* = 0.90, *R^2^* = 0.82, and *p* = 9.94e‐95); and (B) 67‐weeks of age (Pearson's *r* = 0.87, *R^2^* = 0.76, and *p* = 6.79e‐51) in WT and *C9orf72^GR400/+^* mice; and (C) 15‐weeks of age (Pearson's *r* = 0.93, *R^2^* = 0.86, and *p* = 2.42e‐121); (D) 67‐weeks of age (Pearson's *r* = 0.88, *R^2^* = 0.77, *p* = 8.81e‐59) in WT and *Tardbp^Q331K/Q331K^* mice. For number of videos, see Supplementary Table 1; and (E) correlation between two manual scorers of the time spent sniffing each odour presentation (Pearson's *r* = 0.97, *R^2^* = 0.95, and *p* = 2.75e‐92). Total of 16 videos were selected from the *C9orf72* and the *Tardbp* studies, four at each age (15‐ and 67‐weeks of age).

Comparing the time spent sniffing in WT and *C9orf72^GR400/+^* at 15‐ and 67‐weeks of age, we observed a strong and significant positive correlation between manual and ML pipeline determined sniffing time—Pearson's *r* = 0.90, *R^2^* = 0.82, *p* = 9.94e‐95 (Figure 3A), and Pearson's *r* = 0.87, *R^2^* = 0.76, *p* = 6.79e‐51 (Figure 3B), respectively. For the WT and *Tardbp^Q331K/Q331K^* mice, we observed a similar correlation between manual and ML determined sniffing times at both 15‐ and 67‐weeks of age—Pearson's *r* = 0.93, *R^2^* = 0.86, *p* = 2.42e‐121 (Figure 3C), and Pearson's *r* = 0.88, *R^2^* = 0.77, *p* = 8.81e‐59 (Figure 3D). The correlation between two manual scorers is comparable (Figure 3E) to the correlation between ML and manual methods, supporting the efficacy of our ML pipeline for the quantification of sniffing time in the habituation‐dishabituation task.

### 3.2 Comparison of Manual and ML Quantification for the Assessment of Technical and Biological Effects in the Olfactory Habituation‐Dishabituation Task

**FIGURE 4:**
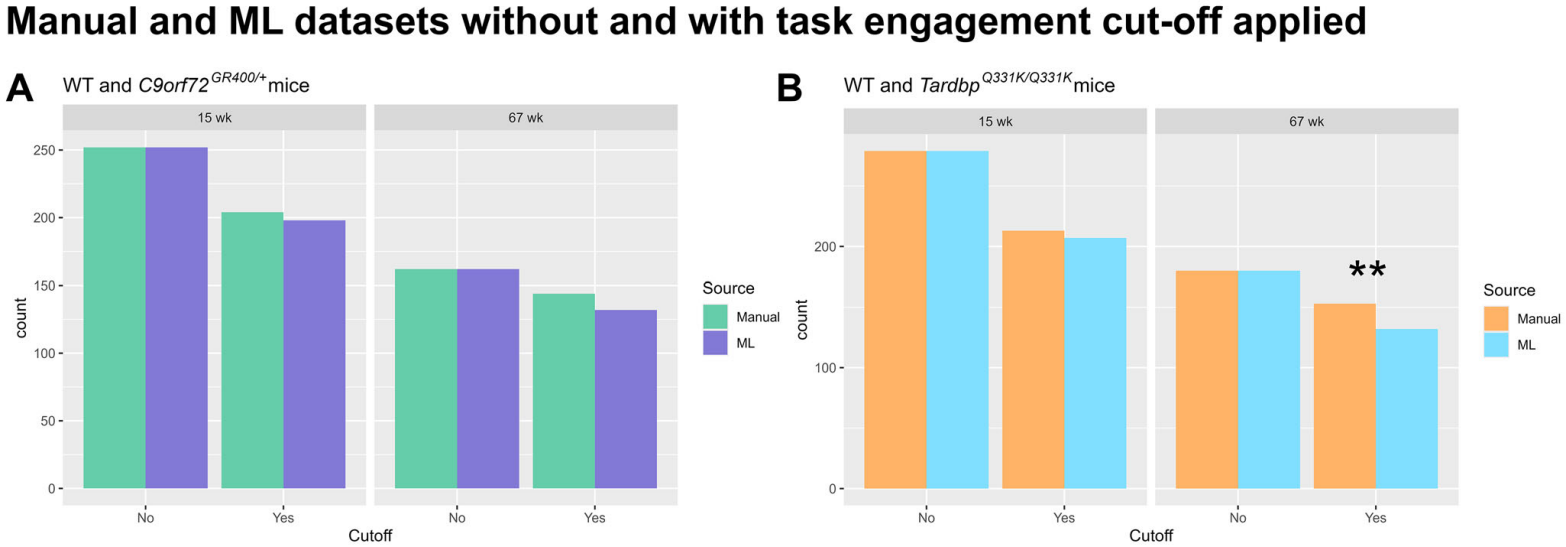
Comparison of data from manual and ML analysis without and with the task engagement cut‐off applied. Histograms showing the number of data points in the datasets without and with task engagement cut‐off applied (trials with total sniffing time <10 s at the first presentation of odour are removed). (A) *C9orf72^GR400/+^* study, green—manual dataset (*n* = 252 cut‐off not applied, *n* = 204 cut‐off applied 15‐weeks of age, *n* = 162 cut‐off not applied, *n* = 144 cut‐off applied 67‐weeks of age), purple—ML dataset (*n* = 252 cut‐off not applied, *n* = 198 cut‐off applied 15 weeks, *n* = 162 cut‐off not applied, *n* = 132 cut‐off applied 67 weeks); and (B) *Tardbp^Q331K/Q331K^* study, orange—manual dataset (*n* = 279 cut‐off not applied, *n* = 213 cut‐off applied 15‐weeks of age, *n* = 180 cut‐off not applied, *n* = 153 cut‐off applied 67 weeks), blue—ML dataset (*n* = 279 cut‐off not applied, *n* = 207 cut‐off applied 15 weeks, *n* = 180 cut‐off not applied, *n* = 132 cut‐off applied 67‐weeks of age). Effect of source of data (manual or ML) and age interaction *χ^2^* = 5.2677, df = 1, *p* = 0.02173, ** *p* < 0.01 post hoc analysis with Bonferroni correction.

To analyse the olfactory habituation‐dishabituation dataset for the biological effects of sex, age, and genotype of animals, trials in which animals did not engage with the task, as indicated by < 10 s sniffing of the first presentation of each odor, are excluded (‘Behavioral Models | Behavioral and Functional Neuroscience Lab | Wu Tsai Neurosciences Institute’ 2023). Therefore, we first determined if our ML pipeline created a bias during this analysis step. We ran a fitted generalised linear mixed model—status of filtering (without or with application of engagement cut‐off for first presentation of odor <10 s) ∼ source (manual or ML) * age with video ID as a random factor. For the *C9orf72* study, we did not observe an effect of source of data (manual or ML) (*χ^2^* = 0.0109, *df* = 1, *p* = 0.91675), age (*χ^2^* = 1.2413, *df* = 1, *p* = 0.26522), or source*age interaction (*χ^2^* = 1.7922, *df* = 1, *p* = 0.18066) on the relative filtering of the data (Figure 4A). However, for the *Tardbp* study, we observed a source of data (manual or ML)*age interaction (*χ^2^* = 5.2677, *df* = 1, *p* = 0.02173) on the filtering of the data based on task engagement. Post hoc analysis using Bonferroni correction showed a difference between engagement status for the 10 s cut‐off for manual and ML data at the 67‐week time point (*p* = 0.0021). This suggests that using the ML output for the old time point in the *Tardbp* study underestimates the level of engagement with the task compared to manual scoring. No main effects of age (*χ^2^* = 2.2131, *df* = 1, *p* = 0.13685) or source of the data (manual or ML) (*χ^2^* = 0.4906, *df* = 1, *p* = 0.48368) on filtering for initial adequate odor engagement were observed in the *Tardbp* study (Figure 4B).

**FIGURE 5:**
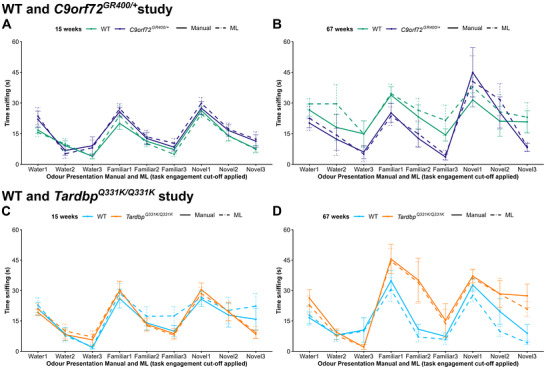
Comparison of the time spent sniffing determined by manual or ML methods by order of odour presentation and genotype with task engagement cut‐off applied. (A–D) Manual scoring (solid line) or the machine learning pipeline (dashed line) generated data of time sniffing each presentation (1–3) of control (water), social familiar and social novel odours, in colony matched WT control (green) and *C9orf72^GR400/+^* (purple), and colony matched WT control (blue) and *Tardp^Q331K/Q331K^* mice (orange), at (A, C) 15 weeks and (B, D) 67‐weeks of age. (A) No effects of data source (manual or ML) *F* (1, 342.90) = 0.0988, *p* = 0.75345, an effect of genotype *F* (1, 25) = 7.3660, *p* = 0.01187, sex *F* (1, 24.73) = 4.4478, *p* = 0.04525, odour type presentation *F* (8, 342.27) = 41.3998, *p* < 2e‐16 was observed at 15‐weeks of age in the *C9orf72^GR400/+^* study; (B) no effect of data source (manual or ML) F (1, 222.863) = 0.9682, *p* = 0.32620, an effect of odour type presentation *F* (8, 223.135) = 14.5512, *p* < 2e‐16, genotype*odour type presentation interaction *F* (8, 223.135) = 2.3644, *p* = 0.01843 was observed at 67‐weeks of age in the *C9orf72^GR400/+^* study; (C) in WT and *Tardp^Q331K/Q331K^* mice at 15 weeks of age, no effect of data source (manual or ML) *F* (1, 356.02) = 2.6738, *p* = 0.1029, an effect of odour type presentation *F* (8, 359.25) = 28.2165, *p* < 2e‐16, and a genotype* odour type presentation interaction *F* (8, 359.25) = 2.3737, *p* = 0.0168 were observed; and (D) in WT and *Tardp^Q331K/Q331K^* mice, at 67 weeks of age, no effect of data source (manual or ML) *F* (1, 232.057) = 3.3788, *p* = 0.06732, an effect of odour type presentation *F* (8, 230.995) = 24.1319, *p* < 2.2e‐16, significant genotype* odour type presentation interaction *F* (8, 230.995) = 4.8783, *p* = 1.41e‐05 was observed. Error bars represent mean ± SEM—standard error mean. Manual and ML: at 15 weeks WT (controls for *C9orf72^GR400/+^*) *n* = 14, Female *n* = 5; *C9orf72^GR400/+^ n* = 14, Female *n* = 8; WT (controls for *Tardp^Q331K/Q331K^*) *n* = 16, Female *n* = 8; *Tardp^Q331K/Q331K^ n* = 15, Female *n* = 6; at 67 weeks WT (controls for *C9orf72^GR400/+^*) *n* = 11, Female *n* = 5; *C9orf72^GR400/+^ n* = 7, Female *n* = 3; WT (controls for *Tardp^Q331K/Q331K^*) *n* = 11, Female *n* = 7 – manual, *n* = 10, Female *n* = 6 – ML; manual and ML *Tardp^Q331K/Q331K^ n* = 9, Female *n* = 3.

To determine if manual compared with ML annotation of sniffing behaviour altered technical or biological outcomes of these olfaction studies, we assessed the data from mice that engaged with the task, and we included only trials that meet the criteria of 10 s or greater initial odor engagement. We studied the effect of data source (manual vs ML) on the time spent interacting with the odor cue across the task by including of data source in a lmer (time spent sniffing ∼ source*genotype*(odor type presentation + sex) + (1|video ID)). We also determined the effects of genotype, sex, and odor type presentation order. In the case of significant main effects, we explored post hoc effects of interest in both sources (manual or ML) to compare the performance of the two pipelines. We found no evidence that data source affected the statistical outcome in either mouse model at either time point in mice that engaged with the task. Moreover, data source did not interact with technical (odor (water, familiar, social) and presentation event (1–9)), or biological (sex, genotype) variables (for full statistical output, see Supplementary Table 2) (Figure 5). Overall, our ML pipeline recapitulated parameters to control for task efficacy and to test our primary hypothesis that mouse genotype affected odor habituation‐dishabituation in mice that engaged with the task.

To further assess the ML pipeline's utility, we undertook post hoc comparisons for any significant main effects observed in the lmer. We particularly assessed odor type presentation order to determine if olfactory habituation and dishabituation were detected, a key technical parameter for the validity of the experiment. We undertook this for every genotype at each age, and source of data (manual or ML) to determine if ML‐generated data resulted in the same technical and biological conclusions as manually annotated data that we previously reported.

For the *C9orf72* study at 15 weeks of age, we detected a main effect of genotype (*F* (1, 25.00) = 7.3660, *p* = 0.01187), odor type presentation (*F* (8, 342.27) = 41.3998, *p* < 2e‐16), and sex (*F* (1, 24.73) = 4.4478, *p* = 0.04525) (Figure 5A). The post hoc comparisons of both the manual and the ML data detected significant habituation to social odors, and significant dishabituation from control to social odors, as well as dishabituation between social odors in both the WT and *C9orf72^GR400/+^* mice. Additionally, manual annotation and the ML pipeline found that the *C9orf72^GR400/+^* mice also showed significant habituation to the control odor and disinhibition from familial social odor to control odor (Supplementary Table 2A). The only post hoc discrepancy between the manual data and our ML pipeline was that the manual scoring showed habituation of the WT mice to the control odor, whereas the ML pipeline did not detect this, at 15‐weeks of age.

For the *C9orf72* study at 67‐weeks of age, we observed a main effect of odor type presentation order *F* (8, 223.135) = 14.5512, *p* < 2e‐16, and an interaction between genotype and odor presentation *F* (8, 223.135) = 2.3644, *p* = 0.01843 (Figure 5B). Post hoc comparisons of both manual annotation and the ML pipeline data failed to detect significant habituation to novel social odor and the control odor in the WT, and to familiar social odor and control in the *C9orf72^GR400/+^* mice, but detected significant habituation to the novel social odor in the *C9orf72^GR400/+^* mice at the later time point. Manual annotation and ML data output successfully detected dishabituation from control to novel social odor, and from familiar to novel social odor in the *C9orf72^GR400/+^* mice, and dishabituation from control to familiar social odor in WT mice. However, at 67‐week‐of age, manual annotation detected significant habituation to the familiar social odor in the WT mice, but this was not detected using the ML pipeline. Whereas the ML pipeline detected dishabituation from control to novel social odor, this was not detected by manual scoring in WT mice (Supplementary Table 2B). Importantly, no evidence of a genotype effect (by post hoc comparison using Bonferroni correction for multiple comparisons) on the duration of sniffing was observed using either manually or ML‐extracted data in the *C9orf72^GR400/+^* study. Thus, ML‐extracted data produced the same overall biological conclusions as manually annotated data in mice that engaged with the task.

In the *Tardbp* study at 15 weeks of age, we detected a main effect of odor type presentation order *F* (8, 359.25) = 28.2165, *p* < 2e‐16 and an interaction between genotype and odor type presentation *F* (8, 359.25) = 2.3737, *p* = 0.0168 (Figure 5C). Post hoc comparisons detected habituation to the control odor in WT mice and to the familiar and the novel social odor in the *Tardbp^Q331K/Q331K^* mice, using data extracted by both manual annotation and the ML pipeline. Similarly, both data extraction methods detected dishabituation from control to the social smells in the WT and *Tardbp^Q331K/Q331K^* mice and dishabituation between the social smells in the *Tardbp^Q331K/Q331K^* mice. The only discrepancies between data sources (manual or ML) were observed in WT mice at the younger time point, in which manual annotation detected habituation to familiar social smell, and dishabituation from familiar to novel smell, whereas the ML pipeline did not (Supplementary Table 2C). No evidence of genotype effect (by post hoc comparison) on duration of sniffing was observed using either manually extracted or ML‐generated data in the *Tardbp* study at the younger time point.

In the *Tardbp* study, at 67‐weeks of age, we detected an effect of odor type presentation *F* (8, 230.995) = 24.1319, *p* < 2.2e‐16 and an interaction between genotype and odor type presentation *F* (8, 230.995) = 4.8783, *p* = 1.41e‐05 (Figure 5D). At 67‐weeks of age, habituation to familiar odor in the WT, and *Tardbp^Q331K/Q331K^* mice and habituation to novel social odor in the WT were detected using our ML pipeline, consistent with previous manual annotation. Similarly, dishabituation from familiar to novel social odor in the WT and *Tardbp^Q331K/Q331K^* mice was detected by ML as previously reported using manual annotation. Also, dishabituation from control to social odors in the *Tardbp^Q331K/Q331K^* mice was detected using both methods of data generation. However, the previously detected, using manual annotation, dishabituation from novel to familiar social odor, and from control to social odors in the WT was not detected in our ML pipeline. Our ML method was also not able to detect habituation to water in the *Tardbp^Q331K/Q331K^* mice but did detect dishabituation between social odors, which was previously not detected using manual analysis. When we explored post hoc comparisons between genotypes, both data extraction methods (manual and ML) showed a significant genotype difference only at the second presentation of familiar social odor (manual *p* = 0.0277, ML *p* = 0.0026), at the later time point in the *Tardbp^Q331K/Q331K^* study (Supplementary Table 2D).

Overall, data extracted using our ML pipeline recapitulated the key parameter to control for task efficacy (odor presentation order) and our primary biological hypothesis to test the effect of an animal's genotype on odor habituation or dishabituation in mice that engaged with the task. However, we found evidence of differences in estimating task engagement, particularly at the 67‐week time‐point in the *Tardbp^Q331K/Q331K^* study, suggesting that the pipeline does not extract behavioral data equally under all conditions, and future optimizations could contribute to improved generalizability.

## 4 Discussion

Here, we developed and validated a customized ML pipeline to efficiently extract data from the mouse olfactory habituation‐dishabituation task, using single camera side‐view videos. We show that freely accessible and widely used ML tools can reach high and significant correlation to manual scoring even in complex home cage environments filmed from the side using a single camera and extract data that leads to similar conclusions to manual annotation methods. This type of video orientation is compatible with long‐term housing of animals under high‐welfare (including group housing) IVC conditions, facilitating the acquisition of longitudinal behavioral data from animals held in the home environment. Moreover, the use of a single camera reduces data complexity compared with multiple camera views and circumvents the need for complex, computationally intensive data integration steps. Indeed, the ML pipeline reported here can be effectively run on a single desktop computer (Nvidia RTX4090 GPU), making it accessible to researchers who do not have access to high‐performance clusters and reducing the energy impact of this type of automated analysis. Also, with this approach, the user benefits from free open‐source packages with higher flexibility compared with the major commercially available software products, and the pipeline can be adapted to include specific behaviors of interest in a complex environment such as an IVC rather than an isolated maze or a non‐IVC cage.

A number of considerations are required for the implementation of this ML pipeline. Technical consistency, particularly the placement and angle of the camera in the rig and the orientation and length of the cotton bud odor cue during data acquisition, are essential for the successful implementation of ML data extraction pipelines. Prior to the start of analysis, both these technical aspects and video data quality control should be undertaken, including the removal of any corrupted or interrupted videos or experiments in which technical errors occurred. Application of the pipeline to new datasets requires a validation step using a subset of manually scored data (ideally by two independent scorers) to determine the efficacy of the pipeline for the new use case and if retraining of either DLC or SimBA will be required. In particular, attention to the manual annotation of ROIs in SimBA for new datasets is required to ensure consistency between users of the pipeline. Additionally, this pipeline, was trained on black C57BL/6J mice, pipeline accuracy may differ if white, beige, or agouti mice are used, and retraining may be required. Similarly, retraining is likely to be needed for technical changes such as different lighting conditions or type and placement of the food hopper. Previously, segmentation networks trained on multiple strains using top‐down view videos have shown good tracking performance across a diverse range of animal appearances (Geuther et al. 2019). In the future, this pipeline could be similarly iterated for use with other strains and technical set‐ups. Furthermore, this pipeline could also be built upon to test olfactory response to different types of smells, such as non‐social odor stimuli or mono‐molecular odors, noting that non‐social odors are known to produce less interest in the animals (Yang and Crawley 2009), which could change patterns of engagement with the task, resulting in a requirement for further optimization. Finally, for mouse strains that have hyperactivity phenotypes, revalidation of the pipeline is recommended since activity level may also alter the pattern of interaction with the task, such that the discrimination threshold and bout length for sniffing events may need adaptation.

This ML pipeline has a number of limitations. Firstly, the ROI annotated in SimBA applies a two‐dimensional projection on three‐dimensional video data, although this is adequate for the task automated in this study, this may confound the efficacy of this tool for other use cases. Our ML pipeline underscored sniffing behavior in the *Tardbp* 67‐week time point and led to underestimation of engagement with the task compared with manual scoring. Thus, our ML pipeline does not extract behavioral task performance data identically under all conditions, and revalidation is recommended prior to implementation in novel datasets. The development of this ML pipeline was enabled by access to two large longitudinal datasets collected under identical technical conditions, which provided a large pool of high‐quality training and test video data. Effective use of pose‐estimation and SimBA for more complex use cases (as reported here) requires adequately powered datasets for train‐test cycles and validation steps, which may not be available to all researchers. In particular, the lack of standardization of data acquisition methods between research groups, and/or small studies may limit the broader utility of these methods. Use of alternative methods of animal tracking, such as segmentation rather than pose estimation may reduce the size of training datasets and partially address this limitation and would be an important avenue for future investigation (Chan et al. 2025).

Specific cases in which our pipeline was found to provide results different from manual scoring included instances where the mouse directly accessed the cotton bud and chewed on it, instances where the mouse was under the food hopper, facing away from the camera when close to the food hopper, and occasions where the inclusion criteria for adequate initial odor exploration differed between animals in the manual and ML datasets. To address these issues, further future improvements for this olfactory habituation‐dishabituation ML pipeline would include developing classifiers that better differentiate between types of interaction with the odor cue (sniffing compared with licking or biting), as well as training a model against datasets assessed by at least two manual scorers to decrease any subjectivity in the manual scoring and improve the quality of the ground truth data. Similarly, these tools can be further developed to classify and quantitate other behaviors in the home‐cage environment observed using a single side‐view infrared camera, enabling additional studies of innate and task‐mediated mouse behavior. In summary, here we demonstrate that efficient ML pipelines can be generated to accurately and effectively automate the quantification of complex behaviors in mice using a single side‐view projection with body part occlusion, enhancing research productivity.

## Author Contributions

**M. H. Correa**: methodology, formal analysis, data curation. **S. Boyanova**: conceptualization, methodology, validation, formal analysis, data curation, writing – original draft, writing – review and editing. **R. S. Bains**: conceptualization, project administration. **F. K. Wiseman**: writing – original draft, writing – review and editing, project administration, conceptualization.

## Funding

F.K.W., and S.B. are supported by the UK Dementia Research Institute (UK DRI Ltd; UKDRI‐1014 and UKDRI‐CIP0202 held by F.W.) through UK DRI Ltd, principally funded by the UK Medical Research Council. F.K.W. was also supported by an Alzheimer's Research UK Senior Research Fellowship (ARUK‐SRF2018A‐001). R.S.B. is supported by the UK Medical Research Council (MC_UP_2201/1, Mary Lyon Centre, International Facility for Mouse Genetics, at MRC Harwell). The funders had no role in study design, data collection and analysis, decision to publish or preparation of the manuscript.

## Ethics Statement

All animal studies were licensed by the Home Office under the Animals (Scientific Procedures) Act 1986 Amendment Regulations 2012 (SI 4 2012/3039), UK, and additionally approved by the Institutional Ethical Review Committee.

## Conflicts of Interest

The authors declare no conflicts of interest. F.K.W. has undertaken for fee consultancy for Alnylam Pharmaceuticals and TRIMTECH Therapeutics.

## Supporting information




**Supplementary Material**: brb371619‐sup‐0001‐TableS1‐S2.docx

## Data Availability

Primary data outputs, genotyping codes, python, and R scripts are available at GitHub: sboya23/ML‐analysis‐olfaction. Other relevant data are included in the article and its supplementary information. Raw data are available from F.K.W. upon reasonable request.
