# Supplementary material for: Using Machine Learning to Automate the Analysis of an Olfactory Habituation‐Dishabituation Task in Mice
Source: Brain Behav. 2026 Jul 28;16(8):e71619. doi: 10.1002/brb3.71619 (PMC13411289; doi:10.1002/brb3.71619)
Supplement: Supplementary file 1 — Supplementary Material: brb371619‐sup‐0001‐TableS1‐S2.docx [file BRB3-16-e71619-s001.docx]

**Supplementary Materials**

**Supplementary Table 1: Number of mice used for the analysis in each test at each time point. Mice were excluded from analysis *only* if they had to be culled for welfare reasons unless otherwise specified.** *2 videos were excluded from any stage of the training and analysis pipeline due to wrong hopper used (67 weeks - C9ORF72-GR400-MAT-B6J/1.3c, C9ORF72-GR400-MAT-B6J/2.2d)*

| WT and *C9orf72^GR400/+^*  Olfaction test | **15 weeks** | **DLC Train (15 videos)**  **WT = 7 (4 female)**  ***C9orf72^GR400/+^* = 8 (3 female)**  **SimBA train (15 DLC videos + 5 additional videos)**  **WT = 10 (7 female)**  ***C9orf72^GR400/+^* = 10 (4 female)** | **67 weeks**  *2 videos excluded due to procedural failure; wrong hopper used (C9ORF72-GR400-MAT-B6J/1.3c, C9ORF72-GR400-MAT-B6J/2.2d)* | **DLC Train (15 videos)**  **WT = 8 (6 female)**  ***C9orf72^GR400/+^* = 7 (4 female)**  **SimBA train (15 DLC videos + 5 additonal videos)**  **WT = 9 (6 female)**  ***C9orf72^GR400/+^* = 11 (6 female)** |
| --- | --- | --- | --- | --- |
|  |  | **Test (28 videos)**  **WT = 14 (5 female)**  ***C9orf72^GR400/+^* = 14 (8 female)** |  | **Test (18 videos)**  **WT = 11 (5 female)**  ***C9orf72^GR400/+^* = 7 (3 female)** |
| WT and *Tardbp^Q331K/Q331K^*  Olfaction test | **15 weeks**  *2 videos which were used for DLC training were excluded from classifiers training – acquisition technical error (wrong order of odours presented during the test) these were valid for training of body pose estimation. (TDP43-Q331K-B6J-IC/1.1c, TDP43-Q331K-B6J-IC/6.1d, at young time point),*  *Mouse TDP43-Q331K-B6J-IC/4.1a was excluded from the final analysis because of a technical error during data acquisition (wrong order of odours presented during the test).* | **DLC Train (15 videos)**  **WT = 8 (4 female)** ***Tardbp^Q331K/Q331K^ =* 7 (5 female)**  **SimBA train (20 videos)**  **WT = 11 (4 female)**  ***Tardbp^Q331K/Q331K^ =* 9 (3 female)** | **67 weeks**  *1 mouse excluded from the test phase analysis due to corrupted video file (TDP43-Q331K-B6J-IC/4.2e)* | **DLC Train (15 videos)**  **WT = 7 (3 female)**  ***Tardbp^Q331K/Q331K^ = 8* (5 female)**  **SimBA train (20 videos)**  **WT = 10 (4 female)** ***Tardbp^Q331K/Q331K^ =* 10 (7 female)** |
|  |  | **Test (31 videos)**  **WT = 16 (8 females)**  ***Tardbp^Q331K/Q331K^ =* 15 (6 female)** |  | **Test (20 videos)**  **WT = 11 (7 female)**  ***Tardbp^Q331K/Q331K^ =* 9 (3 female)** |

**Supplementary Table 2: Full statistical analysis output.** The blue cells represent comparisons relevant to odour habituation, and the purple cells - to odour dishabituation, light and dark for manual and ML, respectively, grey signifies not significant post hoc comparison.

| **A: WT vs *C9orf72^GR400/+^* 15 weeks**  Source – not significant – F (1, 342.90) = 0.0988, p = 0.75345  Genotype F (1, 25) = 7.3660, p = 0.01187  Odour type presentation F (8, 342.27) = 41.3998, p <2e-16  Sex F (1, 24.73) = 4.4478, p = 0.04525 | | | |
| --- | --- | --- | --- |
| Bonferroni correction: pairwise ~ odour type presentation \| genotype \| source  Presenting here only comparisons between first and third odour presentation | | | |
| Mouse group | Odour and presentation comparison | P value (manual) | P value (ML) |
| WT | familiar odour presentation 1 (F1) vs familiar odour presentation 3 (F3) | 0.0137 | <.0001 |
|  | novel odour presentation 1 (N1) vs novel odour presentation 3 (N3) | <.0001 | <.0001 |
|  | water presentation 1 (W1) vs water presentation 3 (W3) | 0.0157 | 0.0757 |
|  | W3 – F1 | 0.0006 | <.0001 |
|  | W3 – N1 | <.0001 | <.0001 |
|  | F3 – N1 | <.0001 | <.0001 |
|  | F3 – W1 | 0.2403 | 0.1570 |
|  | N3 – F1 | 0.0293 | 0.0002 |
|  | N3 – W1 | 0.5041 | 1.0000 |
| *C9orf72^GR400/+^* | F1 – F3 | <.0001 | <.0001 |
|  | N1 – N3 | 0.0001 | <.0001 |
|  | W1 – W3 | 0.0267 | 0.0020 |
|  | W3 – F1 | <.0001 | <.0001 |
|  | W3 – N1 | <.0001 | <.0001 |
|  | F3 – N1 | <.0001 | <.0001 |
|  | F3 – W1 | 0.0185 | 0.0144 |
|  | N3 – F1 | 0.0002 | 0.0002 |
|  | N3 – W1 | 0.1808 | 0.0931 |

| **Bonferroni correction genotype * sex \| source** | **Manual scoring** | **ML scoring** |
| --- | --- | --- |
| Females – contrast for genotype | 0.4374 | 0.0782 |
| Males contrast for genotype | 0.7915 | 0.7339 |
| WT contrast for sex | 0.4301 | 0.3038 |
| *C9orf72^GR400/+^* contrast for sex | 0.9461 | 1.0000 |

| **B: WT vs *C9orf72^GR400/+^* 67 weeks**  Source – not significant – F (1, 222.863) = 0.9682, p = 0.32620  Odour type presentation – F (8, 223.135) = 14.5512, p < 2e-16  Genotype * Odour type presentation – F (8, 223.135) = 2.3644, p = 0.01843 | | | |
| --- | --- | --- | --- |
| Bonferroni correction: pairwise ~ odour type presentation \| genotype \| source  Presenting here only comparisons between first and third odour presentation | | | |
| Mouse group | Odour and presentation comparison | P value (manual) | P value (ML) |
| WT | familiar odour presentation 1 (F1) vs familiar odour presentation 3 (F3) | 0.0190 | 0.6922 |
|  | novel odour presentation 1 (N1) vs novel odour presentation 3 (N3) | 1.0000 | 0.4085 |
|  | water presentation 1 (W1) vs water presentation 3 (W3) | 1.0000 | 0.8635 |
|  | W3 – F1 | 0.0175 | 0.0047 |
|  | W3 – N1 | 0.0701 | 0.0010 |
|  | F3 – N1 | 0.0816 | 0.1942 |
|  | F3 – W1 | 1.0000 | 1.0000 |
|  | N3 – F1 | 0.6863 | 1.0000 |
|  | N3 – W1 | 1.0000 | 1.0000 |
| *C9orf72^GR400/+^* | F1 – F3 | 0.3305 | 0.8283 |
|  | N1 – N3 | 0.0005 | 0.0011 |
|  | W1 – W3 | 1.0000 | 1.0000 |
|  | W3 – F1 | 0.3908 | 0.5313 |
|  | W3 – N1 | <.0001 | 0.0006 |
|  | F3 – N1 | 0.0001 | 0.0009 |
|  | F3 – W1 | 1.0000 | 1.0000 |
|  | N3 – F1 | 1.0000 | 1.0000 |
|  | N3 – W1 | 1.0000 | 1.0000 |

| **Bonferroni correction genotype * odour type presentation \| source Genotype contrast at every odour and source** | **Manual scoring** | **ML scoring** |
| --- | --- | --- |
| Water1 | 1.000 | 1.000 |
| Water 2 | 1.000 | 1.000 |
| Water3 | 1.000 | 1.000 |
| Familiar1 | 1.000 | 1.000 |
| Familiar2 | 1.000 | 1.000 |
| Familiar3 | 1.000 | 0.6330 |
| Novel1 | 1.000 | 1.000 |
| Novel2 | 1.000 | 1.000 |
| Novel3 | 1.000 | 0.8017 |

| **C: WT vs *Tardbp^Q331K/Q331K^* 15 weeks**  Source – not significant – F (1, 356.02) = 2.6738, p = 0.1029  Odour type presentation – F (8, 359.25) = 28.2165, p < 2e-16  Genotype*Odour type presentation – F (8, 359.25) = 2.3737, p = 0.0168 | | | |
| --- | --- | --- | --- |
| Bonferroni correction: pairwise ~ odour type presentation \| genotype \| source  Presenting here only comparisons between first and third odour presentation | | | |
| Mouse group | Odour and presentation comparison | P value (manual) | P value (ML) |
| WT | familiar odour presentation 1 (F1) vs familiar odour presentation 3 (F3) | 0.0034 | 0.3029 |
|  | novel odour presentation 1 (N1) vs novel odour presentation 3 (N3) | 0.6751 | 1.0000 |
|  | water presentation 1 (W1) vs water presentation 3 (W3) | 0.0029 | 0.0004 |
|  | W3 – F1 | <.0001 | <.0001 |
|  | W3 – N1 | <.0001 | <.0001 |
|  | F3 – N1 | 0.0094 | 1.0000 |
|  | F3 – W1 | 1.0000 | 1.0000 |
|  | N3 – F1 | 0.4226 | 1.0000 |
|  | N3 – W1 | 1.0000 | 1.0000 |
| *Tardbp^Q331K/Q331K^* | F1 – F3 | 0.0001 | <.0001 |
|  | N1 – N3 | 0.0001 | 0.0013 |
|  | W1 – W3 | 0.1193 | 0.1636 |
|  | W3 – F1 | <.0001 | <.0001 |
|  | W3 – N1 | <.0001 | 0.0002 |
|  | F3 – N1 | <.0001 | 0.0002 |
|  | F3 – W1 | 0.8627 | 0.2183 |
|  | N3 – F1 | 0.0002 | 0.0002 |
|  | N3 – W1 | 1.0000 | 0.6959 |

| **Bonferroni correction genotype * odour type presentation \| source Genotype contrast at every odour and source** | **Manual scoring** | **ML scoring** |
| --- | --- | --- |
| Water1 | 1.000 | 1.000 |
| Water2 | 1.000 | 1.000 |
| Water3 | 1.000 | 1.000 |
| Familiar1 | 1.000 | 1.000 |
| Familiar2 | 1.000 | 1.000 |
| Familiar3 | 1.000 | 0.6736 |
| Novel1 | 1.000 | 1.000 |
| Novel2 | 1.000 | 1.000 |
| Novel3 | 1.000 | 0.2590 |

| **D: WT vs *Tardbp^Q331K/Q331K^* 67 weeks**  Source – not significant – F (1, 232.057) = 3.3788, p = 0.06732  Odour type presentation – F (8, 230.995) = 24.1319, p < 2.2e-16  Genotype*Odour type presentation – F (8, 230.995) = 4.8783, p = 1.41e-05 | | | |
| --- | --- | --- | --- |
| Bonferroni correction: pairwise ~ odour type presentation \| genotype \| source  Presenting here only comparisons between first and third odour presentation | | | |
| Mouse group | Odour and presentation comparison | P value (manual) | P value (ML) |
| WT | familiar odour presentation 1 (F1) vs familiar odour presentation 3 (F3) | 0.0012 | 0.0168 |
|  | novel odour presentation 1 (N1) vs novel odour presentation 3 (N3) | 0.0020 | 0.0322 |
|  | water presentation 1 (W1) vs water presentation 3 (W3) | 1.0000 | 1.0000 |
|  | W3 – F1 | 0.0071 | 0.1796 |
|  | W3 – N1 | 0.0092 | 0.1551 |
|  | F3 – N1 | 0.0014 | 0.0155 |
|  | F3 – W1 | 1.0000 | 1.0000 |
|  | N3 – F1 | 0.0023 | 0.0513 |
|  | N3 – W1 | 1.0000 | 1.0000 |
| *Tardbp^Q331K/Q331K^* | F1 – F3 | 0.0001 | <.0001 |
|  | N1 – N3 | 1.0000 | 0.6950 |
|  | W1 – W3 | 0.0203 | 0.1184 |
|  | W3 – F1 | <.0001 | <.0001 |
|  | W3 – N1 | <.0001 | <.0001 |
|  | F3 – N1 | 0.0340 | 0.0215 |
|  | F3 – W1 | 1.0000 | 1.0000 |
|  | N3 – F1 | 0.1120 | 0.0064 |
|  | N3 – W1 | 1.0000 | 1.0000 |

| **Bonferroni correction genotype * odour type presentation \| source Genotype contrast at every odour and source** | **Manual scoring** | **ML scoring** |
| --- | --- | --- |
| Water1 | 1.0000 | 1.0000 |
| Water2 | 1.0000 | 1.0000 |
| Water3 | 0.8011 | 1.0000 |
| Familiar1 | 1.0000 | 0.3506 |
| Familiar 2 | 0.0277 | 0.0026 |
| Familiar 3 | 1.0000 | 1.0000 |
| Novel1 | 1.0000 | 1.0000 |
| Novel2 | 1.0000 | 0.3316 |
| Novel3 | 0.3195 | 0.5694 |
